# Understanding the rise in drug mortality rates among black Americans in the 2010s: Associations with county-level construction job shares in the United States

**DOI:** 10.1016/j.pmedr.2025.103146

**Published:** 2025-06-22

**Authors:** Sehun Oh, Manuel Cano, Trenette Clark Goings

**Affiliations:** a College of Social Work, The Ohio State University, Columbus, OH, United States; b School of Social Work, Arizona State University, Phoenix, AZ, United States; c School of Social Work, The University of North Carolina, Chapel Hill, NC, United States

**Keywords:** Black/African American, Construction job share, Drug epidemic, Mortality, U.S. counties

## Abstract

**Objective:**

In 2020, Black Americans became one of the racial/ethnic groups with the highest drug mortality rates, yet factors driving this trend remain unclear. Given the increasing participation of Black workforce in the construction sector during the 2010s—a sector that typically offers livable wages without requiring college degrees—this study examines whether changes in the concentration of local construction sector jobs are associated with variations in drug mortality among Black Americans across U.S. counties.

**Methods:**

Data were drawn from the National Center for Health Statistics' Multiple Cause of Death file, linked with construction job shares, drug supply, and sociodemographic characteristics from other administrative data sources. After examining county characteristics based on changes in construction job shares from 2010–2013 to 2018–2021, first-difference regression models assessed the effects of construction job shares on drug mortality rates among overall and working-aged Black populations.

**Results:**

A 1 percentage point increase in construction job shares was associated with a reduction of 6.67 drug-related deaths per 100,000 among Black residents, with a larger effect (10.32 deaths) among working-aged individuals. Over the study period, this translates to a shift in drug mortality rates from an increase of 38.2 deaths per 100,000 in counties with a 0.5 percentage point decline in construction job shares to a decrease of 1.8 deaths per 100,000 in counties with a 5.5 percentage point increase.

**Conclusions:**

Growing employment opportunities in the construction sector may provide population-level protection against drug mortality for Black Americans, particularly among the working-aged population.

## 1 Introduction

In 2020, Black Americans became one of the racial/ethnic groups with the highest drug mortality rates, following a decade of disproportionately increasing rates (Kariisa et al., 2022). However, this troubling trend was not uniform across the United States, with Black Americans in lower-income counties in the Midwest and Northeast most affected (McLean, 2016). While the underlying drivers remain unclear, a recent study suggests that local employment context may provide valuable insights. Specifically, an increase of one job per 100 Black workers in a county was associated with 0.29 fewer drug overdose deaths per 100,000 Black residents (Oh and Cano, 2024). These findings align with the “disease of despair” perspective (Case and Deaton, 2017), which posits that disconnection from work can lead to various neighborhood disorders and psychological distress, ultimately increasing drug use as a coping mechanism (Dasgupta et al., 2018; Monnat, 2019; Rudolph et al., 2020).

While increasing employment opportunities can offer significant preventive benefits, not all jobs provide equal benefits or accessibility, particularly for minoritized populations. Among various industries, the construction sector warrants special attention in understanding drug mortality for Black Americans during the 2010s. Following the Great Recession, we observed notable employment growth in the construction sector, at twice the rate of overall workforce expansion (U.S. Bureau of Labor Statistics, 2020a; U.S. Bureau of Labor Statistics, 2020b). Moreover, despite being historically over-represented by white workers, the construction sector saw rapid growth in Black workers, increasing by 48.5 % in less than a decade (U.S. Bureau of Labor Statistics, 2020a; U.S. Bureau of Labor Statistics, 2020b). Construction jobs are particularly appealing because they typically offer livable wages—average weekly gross earnings of $1510 in 2024 versus $1209 across all private sectors (US Bureau of Labor Statistics, 2024)—often without requiring a college degree, making them accessible to individuals facing financial and time constraints. As a result, counties experiencing greater growth in the construction sector may provide increased access to meaningful employment for Black workers with socioeconomic barriers, fostering economic mobility. Additionally, improved wages and job stability can strengthen the socioeconomic fabric of communities, enhancing psychological and social resources. These benefits may, in turn, positively influence substance use behaviors, even among residents not directly employed in the sector (Monnat, 2019).

On the other hand, research also highlights the potential adverse effects of increased construction employment on drug mortality. While evidence specific to Black individuals remains limited, studies—particularly those examining decedent-level prevalence—indicate a heightened risk of drug mortality among construction workers. According to Billock et al. (2023) drug mortality rates in the construction sector were 130.9 deaths per 100,000 workers in 2020, three times higher than the rate across all industries combined (41.3 deaths). Construction workers are disproportionately affected by musculoskeletal disorders from workplace injuries and physical strain, often leading to treatment and/or self-treatment with pain medication and thereby increasing the risk of opioid misuse and overdose (Dale et al., 2021; Dong et al., 2022; U.S. Bureau of Labor Statistics, 2021).

Given these mixed effects, it remains an empirical question whether a locality, such as a county, with a higher concentration in the construction sector exerts an overall preventive or contributory impact. Addressing this question requires comparing localities with varying jobs shares in construction, which introduces the potential for bias due to other local-specific determinants of drug mortality correlated with local labor market contexts. To mitigate potential endogeneity, this study employs a first-difference regression approach, leveraging longitudinal county-level data on drug mortality and construction job shares.

## 2 Methods

### 2.1 Data and sample

County-level drug mortality data were obtained from the National Center for Health Statistics' Multiple Cause of Death file, which compiles death certificate information from U.S. Vital Statistics jurisdictions. Drug-related overdose deaths of any intent were identified using the International Classification of Disease-10 codes: X40–44 (unintentional drug poisoning), X60–64 (intentional drug poisoning), X85 (drug poisoning homicide), or Y10–14 (drug poisoning of undetermined intent). For reliable county-level estimates of drug mortality rates among non-Hispanic Black populations, we pooled four years of mortality data (2010–2013 and 2018–2021). These data were then linked with four additional sources: U.S. Census' Longitudinal Employer-Household Dynamics Origin-Destination Employment Statistics (for county-level job shares for 20 industry sectors based on the 2-digit codes of the North American Industry Classification System), CDC (for county-level opioid prescription rates), U.S. Drug Enforcement Administration's National Forensic Laboratory Information System (for state-level fentanyl seizure rates), and American Community Surveys (for county-level sociodemographic characteristics). Given the CDC's data suppression of counties with fewer than 10 deaths, our analytic sample was restricted to 214 counties that reported at least 10 overdose deaths among Black residents during the study periods. These counties are larger and have more populous Black communities compared to excluded counties (Table A.1), accounting for over 82 % of all drug-related overdose deaths among Black Americans during the study period. This study is classified as non-human subjects research and therefore does not require ethical approval.

### 2.2 Measures

First-differences (value in 2018–2021 minus value in 2010–2013) of the following measures were computed for our first-difference regression models.

*Drug mortality rates.* The outcome variable is each county's drug mortality rate per 100,000 Black residents.

*Construction job shares.* The independent variable is a county's ratio of the number of jobs in construction to the total jobs, in percentage form (multiplied by 100). Job shares of the other 19 industries will also be examined as control variables for supplemental analyses.

*Controls.* Controls included sociodemographic (e.g., percentages of Black population, males, individuals ages 25+ without a high school diploma and veterans; median household income; region) and drug supply (opioid prescribing rates; fentanyl seizure rates) characteristics.

### 2.3 Statistical analysis

Statistical analyses were conducted in three steps. First, we examined county characteristics across terciles based on changes in construction job shares from 2010–2013 to 2018–2021. Second, we estimated first-difference regression models to assess the association between construction job shares and drug mortality rates among Black residents, separately for all ages and working ages (ages 15–64). In addition to controlling for sociodemographic and drug supply characteristics, our first-difference regression models account for time-constant unobserved confounders, reducing estimation biases (Wooldridge, 2010). As a supplemental analysis, we also estimated first-difference regression models incorporating job shares from other industries (with education services serving as the reference given their low drug overdose deaths risks) to control for the potential influences of varying county-level industry compositions. Finally, we computed predicted mean changes in drug mortality rates for Black individuals, separately for those of all ages and working ages, across the observed construction job share levels during the study period. Analyses were conducted using Stata/MP v.18.0.

## 3 Results

**Fig. 1.**
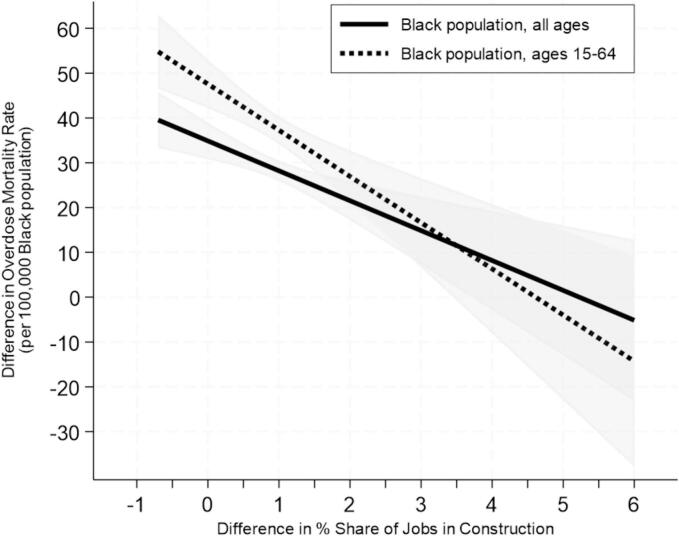
Predicted Mean Changes in Drug Overdose Mortality Rates for the Black Population in the United States across Observed Changes in Job Shares in the Construction Sector from 2010–2013 to 2018–2021. *Notes*. The predicted changes in the drug overdose rates were estimated using the coefficients in Table 1. Drug mortality rates among the Black population are per 100,000 individuals. The difference in job shares in the construction sector was calculated by subtracting the job shares from 2010–2013 from those in 2018–2021 for each included county. The x-axis range reflects the actual changes in job shares in the construction sector across the 214 U.S. counties from 2010–2013 to 2018–2021, ranging from −0.72 % to 6.04 %. The predicted probabilities were estimated at the means of the covariates.

Counties with larger increases in construction job shares—primarily Southern and Western states with fewer Black populations, higher median household incomes, and lower fentanyl seizure rates—experienced smaller increases in drug mortality among Black residents (Table A.2). The results of our first-difference regression analysis in Table 1 reveal that a 1 percentage point increase in construction job share from 2010–2013 to 2018–2021 was associated with a decrease of 6.67 drug overdose deaths per 100,000 among Black residents, with a greater reduction among the working-aged individuals (*b* = 10.32, *p* < .001). These coefficients can be translated into the estimated changes in the drug mortality rates over the study period for the overall Black population ranged from an increase of 38.2 deaths per 100,000 in counties where the construction job share decreased by 0.5 percentage point to a decrease of 1.8 deaths per 100,000 in counties where the construction job share increased by 5.5 percentage point (Fig. 1). For the working-aged Black population, the estimated changes ranged from an increase of 52.7 deaths per 100,000 to a decrease of 9.2 deaths, respectively.

**Table 1.** First-Difference Estimators Predicting the Effects of Job Shares in the Construction Sector on the Changes in the Drug Overdose Mortality Rates among Non-Hispanic Black Populations in the United States from 2010–-2013 to 2018–2021.

	Drug Overdose Mortality Rates for All Ages (*n* = 214)			Drug Overdose Mortality Rates for Working Ages (*n* = 207)		
	*b*	*SE*	*Beta*	*b*	*SE*	*Beta*
∆ Job Shares in Construction	−6.67^⁎⁎⁎^	1.79	−0.26	−10.32^⁎⁎⁎^	2.34	−0.30
						
**Sociodemographic Characteristics**						
∆ Percent Black Population	−1.85	1.45	−0.09	−4.45^⁎^	1.79	−0.17
∆ Percent Male	1.47	6.56	0.02	−0.81	8.68	−0.01
∆ Percent Age 65+	3.21	3.08	0.08	3.10	3.63	0.06
∆ Percent Age 25+ w/o high school	−2.81	1.43	−0.14	-4.41	1.93	−0.17
∆ Percent Veteran	1.66	2.83	0.04	0.18	3.93	0.01
∆ Percent Vacant housing	−0.70	0.61	−0.07	-0.36	0.84	−0.03
∆ Percent Unemployment rate	−1.01	0.87	−0.08	-0.66	1.21	−0.04
∆ Median household Income (in $1000 s)	0.07	0.29	0.02	-0.13	0.40	−0.02
Region						
Midwest (Reference)	–			–		
Northeast	1.25	3.84	0.03	-2.14	5.21	−0.03
South	−6.18^⁎⁎^	3.83	−0.15	-5.58	4.98	−0.11
West	3.70	5.54	0.07	3.48	7.08	0.05
**Drug-related Characteristics**						
∆ County opioid prescribing rate (per 100 persons)	−0.15^⁎^	0.11	−0.11	-0.27	0.14	−0.15
∆ State fentanyl seizures (per 100,000 persons)	0.08^⁎⁎^	0.03	0.21	0.13^⁎⁎^	0.04	0.25

*Notes*. *SE* = standard error. ∆ = difference between the value in 2018–2021 and the value in 2010–2013. The analytic sample consists of counties with at least 10 drug-involved overdose deaths among non-Hispanic Black residents in 2010–2013 and 2018–2021, i.e., 214 counties for drug overdose mortality rates for all ages and 207 counties for drug overdose mortality rates for working-ages (15–64). Mortality rates are per 100,000 standard population. All models also include age composition variables for the Black population (percent ages 0–14, 15–24, 25–44, 45–64) to account for potential variations in age distribution. The coefficients for age composition variables are not presented to conserve space. *p < .05. **p < .01. ***p < .001.

Supplemental analysis controlling for the composition of other industries shows similar patterns, with larger coefficients for both all ages and working ages. Specifically, a 1 percentage point increase in construction jobs is associated with a decrease of 9.61 drug overdose deaths per 100,000 among Black residents of all ages and 12.39 drug overdose deaths per 100,000 among Black residents aged 15–64 (Table A.3). These estimates suggest predicted mean changes in drug mortality rates: an increase of 42.5 deaths per 100,000 (55.8 for ages 15–64) in counties experiencing a 0.5 percentage point reduction in construction job share, and a decrease of 15.2 deaths per 100,000 (18.6 for ages 15–64) in counties experiencing a 5.5 percentage point increase in construction job share (Fig. A.1).

## 4 Discussion

Our findings indicate that drug mortality rates among Black Americans in the 2010s were highest in counties with little to no increases in construction job shares. These counties were primarily located in the South and Northeast, with larger Black populations, low household incomes, and potentially higher circulation of illicitly-manufactured fentanyl. While overall drug mortality has been pronounced in the Midwest and Northeast counties (National Cener for Health Statistics, 2025), our findings underscore the importance of also focusing on Southern counties with larger Black populations, where minimal growth in construction job shares has been observed.

Second, while decedent-level studies have reported higher drug mortality rates among individuals in the construction sector, our ecological models—accounting for county's time-constant unobserved heterogeneity—suggest a protective effect associated with higher construction job shares. Specifically, our findings indicate that each 1 % increase in construction job share is associated with reductions of 6.67 and 10.32 drug overdose deaths per 100,000 among all-aged and working-aged Black Americans, respectively. Prior research consistently highlights that expanding employment opportunities is a critical protective factor against drug-related deaths (Betz and Jones, 2018; Oh and Cano, 2024). Our findings further suggest that increasing opportunities in the construction sector, which typically offer competitive pay for workers without bachelor's degrees, offers overall preventive benefits for Black populations at the county level. However, given the elevated risk of drug-related mortality among construction workers, expanding job opportunities in this sector must be accompanied by evidence-based workplace policies, harm reduction strategies, and support services that address substance use risks. Without such safeguards, the potential health benefits of employment growth in construction may not be fully realized (Oh, 2023).

These findings should be interpreted with caution. First, our data on county-level construction job share changes do not necessarily reflect the actual employment opportunities Black workers face in the sector. Although we observe rapid growth in the proportion of Black workers in the construction sector during the 2010s, their experiences may vary depending on each county's labor market conditions and hiring practices. Second, our first-difference regression models may still be subject to other sources of bias. While this study controlled for a variety of time-varying sociodemographic and substance-related determinants of drug mortality, other local factors that could correlate with changes in construction job shares (e.g., population dynamics; economic development policies impacting other industry sectors) may have influenced our estimates. Third, the overall effects may have been overestimated by including 15-year-olds, who are unlikely to work in the construction sector. However, any impact is likely minimal given their low risk of drug-related mortality. Finally, the results may not be generalizable beyond the 214 counties with at least 10 drug overdose deaths in Black Americans during the study periods, especially counties with smaller Black populations, where reliable drug mortality data are limited.

Despite its limitations, this study provides valuable population-level insights into how local labor market dynamics—particularly in the construction sector—may have contributed to the sharp rise in drug mortality among Black Americans during the 2010s. The increasing and disproportionate risks of drug mortality among Black Americans call for a closer examination of local employment opportunities, especially the South and Midwest, where construction job growth has been relatively stagnant. Expanding job opportunities in this sector—alongside targeted workforce training programs aimed at increasing Black workforce representation—may help mitigate the rising trend in drug mortality, particularly among working-aged Black Americans. Furthermore, it is essential that both research and policy responses explicitly account for structural barriers within local labor markets (e.g., discriminatory hiring practices; limited access to job training; unequal employment protection) that shape who benefits from the expansion. Without addressing these structural barriers, expanding job opportunities in the construction sector alone is unlikely to reduce drug-related harms in historically marginalized communities.

## CRediT authorship contribution statement

**Sehun Oh:** Writing – original draft, Investigation, Data curation, Methodology, Funding acquisition, Conceptualization. **Manuel Cano:** Software, Data curation, Writing – review & editing, Formal analysis, Conceptualization. **Trenette Clark Goings:** Conceptualization, Writing – review & editing.

## Funding

This work was supported by the 10.13039/100000026National Institute on Drug Abuse [Award number: K01DA057514]. The content is solely the responsibility of the authors and does not necessarily represent the official views of NIDA or the NIH.

## Declaration of competing interest

The authors declare that they have no known competing financial interests or personal relationships that could have appeared to influence the work reported in this paper.

## Appendix A Supplementary data

The supplemental materials include a description of the characteristics of counties included and excluded from the analyses; sociodemographic and substance-related characteristics of included counties by terciles of change in construction job share; and results from first-differenced estimators and predicted mean changes from a model adjusting for all other industry sectors. Image 1
