# Supplementary material for: Understanding the rise in drug mortality rates among black Americans in the 2010s: Associations with county-level construction job shares in the United States
Source: Prev Med Rep. 2025 Jun 22;56:103146. doi: 10.1016/j.pmedr.2025.103146 (PMC12246689; doi:10.1016/j.pmedr.2025.103146)
Supplement: Supplementary file 1 — The supplemental materials include a description of the characteristics of counties included and excluded from the analyses; sociodemographic and substance-related characteristics of included counties by terciles of change in construction job share; and results from first-differenced estimators and predicted mean changes from a model adjusting for all other industry sectors. [file mmc1.docx]

**Table A.1**

*Sociodemographic and Substance-Related Characteristics of 214 US Counties, by Tercile of Job Share Changes in Construction Sector from 2010–2013 to 2018–2021*

| **Sociodemographic characteristic** | **2010–2013** | | **2018–2021** | | **∆** | **∆%** |
| --- | --- | --- | --- | --- | --- | --- |
|  | **Mean** | ***SD*** | **Mean** | ***SD*** |  |  |
| **Lowest tercile** |  |  |  |  |  |  |
| Construction job share (%) | 3.70 | 1.25 | 4.09 | 1.22 | 0.39^***^ | 10.51 |
| Total number of jobs | 274,794 | 296,181 | 295,548 | 316,435 | 20,754^***^ | 7.55 |
| Total number of jobs in construction | 8,970 | 7,938 | 10,917 | 9,986 | 1,947^***^ | 21.70 |
| Population size (1000s) | 650.68 | 701.95 | 669.68 | 709.38 | 19.00^***^ | 2.92 |
| Black (%) | 24.53 | 15.14 | 24.72 | 14.87 | 0.18 | 0.75 |
| Male (%) | 48.50 | 0.88 | 48.54 | 0.93 | 0.04 | 0.09 |
| Age 65+ (%) | 12.48 | 2.05 | 14.61 | 2.21 | 2.12^***^ | 17.02 |
| Age 25+ without high school (%) | 12.66 | 4.09 | 10.72 | 3.51 | -1.94^***^ | -15.31 |
| Veteran (%) | 9.19 | 3.71 | 7.54 | 3.67 | -1.65^***^ | -17.92 |
| Vacant housing (%) | 4.71 | 2.87 | 3.32 | 2.66 | -1.40^***^ | -29.65 |
| Unemployment rate (%) | 9.42 | 2.21 | 5.81 | 1.27 | -3.61^***^ | -38.29 |
| Median household income ($) | 54,453 | 14,863 | 62,893 | 16,949 | 8,440^***^ | 15.50 |
| Region (%) |  |  |  |  |  |  |
| Midwest | 18.06 |  |  |  |  |  |
| Northeast | 26.39 |  |  |  |  |  |
| South | 54.17 |  |  |  |  |  |
| West | 1.39 |  |  |  |  |  |
| Opioid prescription per 100 population | 88.64 | 36.63 | 54.42 | 22.40 | -34.22^***^ | -38.61 |
| State fentanyl seizures per 100,000 population | 0.27 | 0.19 | 41.86 | 45.03 | 41.59^***^ | 15544.16 |
| NH Black drug mortality rate per 100,000 population | 10.76 | 5.86 | 44.63 | 27.45 | 33.87^***^ | 314.71 |
| **Middle tercile** |  |  |  |  |  |  |
| Construction job share (%) | 3.77 | 1.23 | 4.54 | 1.26 | 0.78^***^ | 20.60 |
| Total number of jobs | 297,082 | 255,924 | 331,231 | 293,729 | 34150^***^ | 11.50 |
| Total number of jobs in construction | 11,409 | 14,185 | 15,270 | 18,270 | 3,861^***^ | 33.84 |
| Population size (1000s) | 695.32 | 623.51 | 727.96 | 675.75 | 32.64^***^ | 4.69 |
| Black (%) | 17.55 | 12.73 | 17.87 | 13.05 | 0.32^*^ | 1.83 |
| Male (%) | 48.75 | 0.74 | 48.80 | 0.77 | 0.05^*^ | 0.10 |
| Age 65+ (%) | 12.63 | 2.54 | 14.78 | 2.69 | 2.14^***^ | 16.97 |
| Age 25+ without high school (%) | 12.19 | 4.21 | 10.57 | 3.78 | -1.62^***^ | -13.32 |
| Veteran (%) | 8.56 | 2.91 | 6.94 | 2.99 | -1.62^***^ | -18.95 |
| Vacant housing (%) | 4.26 | 2.12 | 3.10 | 2.18 | -1.16^***^ | -27.16 |
| Unemployment rate (%) | 9.70 | 2.44 | 5.34 | 1.40 | -4.37^***^ | -45.00 |
| Median household income ($) | 58,581 | 16,038 | 68,848 | 19,900 | 10,267^***^ | 17.53 |
| Region (%) |  |  |  |  |  |  |
| Midwest | 39.44 |  |  |  |  |  |
| Northeast | 26.76 |  |  |  |  |  |
| South | 28.17 |  |  |  |  |  |
| West | 5.63 |  |  |  |  |  |
| Opioid prescription per 100 population | 80.82 | 25.86 | 49.78 | 16.51 | -31.04^***^ | -38.40 |
| State fentanyl seizures per 100,000 population | 0.24 | 0.18 | 54.83 | 64.42 | 54.59^***^ | 22318.61 |
| NH Black drug mortality rate per 100,000 population | 12.25 | 8.67 | 42.65 | 27.76 | 30.40^***^ | 248.15 |
| **Highest tercile** |  |  |  |  |  |  |
| Construction job share (%) | 4.45 | 1.17 | 6.16 | 1.69 | 1.71^***^ | 38.37 |
| Total number of jobs | 406,391 | 530,057 | 465,805 | 600,897 | 59,415^***^ | 14.62 |
| Total number of jobs in construction | 16,643 | 17,801 | 26,102 | 28,193 | 9,460^***^ | 56.84 |
| Population size (1000s) | 997.67 | 1,324.80 | 1,059.77 | 1,375.51 | 62.11^***^ | 6.23 |
| Black (%) | 14.45 | 12.47 | 14.64 | 12.50 | 0.20 | 1.35 |
| Male (%) | 49.10 | 0.79 | 49.12 | 0.80 | 0.01 | 0.02 |
| Age 65+ (%) | 13.12 | 3.38 | 15.37 | 3.49 | 2.25^***^ | 17.17 |
| Age 25+ w/o high school (%) | 13.63 | 4.66 | 11.70 | 4.31 | -1.93^***^ | -14.19 |
| Veteran (%) | 9.56 | 2.97 | 7.82 | 2.87 | -1.74^***^ | -18.17 |
| Vacant housing (%) | 6.06 | 2.77 | 5.10 | 2.89 | -0.96^***^ | -15.87 |
| Unemployment rate (%) | 10.64 | 2.28 | 5.57 | 1.37 | -5.07^***^ | -47.68 |
| Median household income ($) | 57,747 | 13,499 | 69,090 | 17,010 | 11,342^***^ | 19.64 |
| Region (%) |  |  |  |  |  |  |
| Midwest | 11.27 |  |  |  |  |  |
| Northeast | 12.68 |  |  |  |  |  |
| South | 40.85 |  |  |  |  |  |
| West | 35.21 |  |  |  |  |  |
| Opioid prescription per 100 population | 82.82 | 25.85 | 51.28 | 17.63 | -31.54^***^ | -38.08 |
| State fentanyl seizures per 100,000 population | 0.18 | 0.17 | 26.71 | 39.98 | 26.53^***^ | 15159.90 |
| NH Black drug mortality rate per 100,000 population | 11.25 | 5.43 | 32.44 | 13.17 | 21.19^***^ | 188.33 |

*Notes*. Drug mortality rates are Black Americans’ drug overdose deaths per 100,000 individuals.

*SD* = standard deviation

∆% = difference between value in 2018–2021 and value in 2010–2013, divided by the value in 2010-2013, and finally multiplied by 100.

*p* values are for the difference between 2010–2013 and 2018–2021 values, via paired *t*-tests

**p* < .05. ***p* < .01. ****p* < .001

**Table A.2**

*First-Difference Estimators Predicting the Effects of Job Shares in Construction Sector on the Changes in the Drug Overdose Mortality Rates among Non-Hispanic Black Populations from 2010–2013 to 2018–2021: Controlling for Job Shares in All Industry Sectors*

|  | Drug Overdose Mortality Rates for All Ages  (*n* = 214) | | | Drug Overdose Mortality Rates for Working Ages  (*n* = 207) | | |
| --- | --- | --- | --- | --- | --- | --- |
|  | *b* | *SE* | *Beta* | *b* | *SE* | *Beta* |
| ∆ Job Shares in Construction | −9.61^**^ | 2.80 | −0.37 | -12.39^**^ | 3.77 | -0.36 |
|  |  |  |  |  |  |  |
| **Sociodemographic Characteristics** |  |  |  |  |  |  |
| ∆ Percent Black Population | −2.53 | 1.51 | −0.13 | -4.38^*^ | 1.87 | -0.17 |
| ∆ Percent Male | 1.74 | 6.78 | 0.02 | 0.38 | 8.94 | 0.01 |
| ∆ Percent Age 65+ | 4.46 | 3.16 | 0.11 | 3.61 | 3.69 | 0.07 |
| ∆ Percent Age 25+ w/o high school | −2.32 | 1.53 | −0.12 | -3.89 | 2.06 | -0.15 |
| ∆ Percent Veteran | 0.56 | 2.85 | 0.01 | -0.78 | 3.95 | -0.01 |
| ∆ Percent Vacant housing | −0.31 | 0.62 | −0.03 | -0.18 | 0.85 | -0.01 |
| ∆ Percent Unemployment rate | −0.70 | 1.00 | −0.06 | -0.17 | 1.36 | -0.01 |
| ∆ Median household Income (in $1,000s) | −0.69 | 0.45 | −0.17 | -1.27^*^ | 0.60 | -0.24 |
| Region |  |  |  |  |  |  |
| Midwest (Reference) | - | | |  | | |
| Northeast | 2.91 | 4.66 | 0.06 | 0.81 | 6.28 | 0.01 |
| South | −0.92^**^ | 4.32 | −0.02 | -0.78 | 5.60 | -0.01 |
| West | 0.58 | 6.62 | 0.01 | -3.05 | 8.86 | -0.04 |
| **Drug-related Characteristics** |  |  |  |  |  |  |
| ∆ Opioid prescribing rate (per 100 persons) | −0.12 | 0.11 | −0.09 | -0.15 | 0.14 | -0.08 |
| ∆ State fentanyl seizures (per 100,000 persons) | 0.09^**^ | 0.03 | 0.24 | 0.12^**^ | 0.04 | 0.24 |

*Notes*. *SE* = standard error. ∆ = difference between the value in 2018–2021 and the value in 2010–2013. First-differenced regression models were estimated separately for drug overdose death rates among Black individuals of all ages and working-ages, controlling for the same control variables as in the main analyses (Table 1) and job share variables for all other industry sectors, with educational services as the reference group. The analytic sample consists of counties with at least 10 drug-involved overdose deaths among non-Hispanic Black residents in 2010–2013 and 2018-2021. Mortality rates are per 100,000 standard population. All models also include age composition variables for the Black population (percent ages 0-14, 15-24, 25-44, 45-64) to account for potential variations in age distribution. The coefficients for age composition and other industries’ job share variables are not presented to conserve space. **p* < .05. ***p* < .01. ****p* < .001

**Figure A.1**

*Predicted Mean Changes in Drug Overdose Mortality Rates for the Black Population across Observed Changes in Job Shares in the Construction Sector from 2010–2013 to 2018–2021*

*Notes*. The predicted changes in the drug overdose rates were estimated using coefficients from the first-differenced regression models that included job shares from all industry sectors, with educational services as the reference group. Drug mortality rates among the Black population are per 100,000 individuals. The difference in job shares in the construction sector was calculated by subtracting the job shares from 2010–2013 from those in 2018–2021 for each included county. The x-axis range reflects the actual changes in job shares in the construction sector across the 214 U.S. counties from 2010–2013 to 2018–2021, ranging from -0.72% to 6.04%. The predicted probabilities were estimated at the means of the covariates.
